# Rubisco carboxylation kinetics and inorganic carbon utilization in polar versus cold-temperate seaweeds

**DOI:** 10.1093/jxb/ery443

**Published:** 2018-12-21

**Authors:** Concepción Iñiguez, Jeroni Galmés, Francisco J L Gordillo

**Affiliations:** 1 Department of Ecology, Faculty of Sciences, University of Malaga, Boulevard Louis Pasteur s/n, Málaga, Spain; 2 Research Group in Plant Biology under Mediterranean Conditions, Universitat de les Illes Balears-INAGEA, Carretera de Valldemossa, Palma, Illes Balears, Spain

**Keywords:** Carbon concentrating mechanisms, carbon fixation, kinetics, macroalgae, photosynthesis, polar, Rubisco, seaweeds

## Abstract

Despite the high productivity and ecological importance of seaweeds in polar coastal regions, little is known about their carbon utilization mechanisms, especially the kinetics of the CO_2_-fixing enzyme Rubisco. We analyzed Rubisco carboxylation kinetics at 4 °C and 25 °C in 12 diverse polar seaweed species (including cold-temperate populations of the same species) and the relationship with their ability to use bicarbonate, by using ^13^C isotope discrimination and pH drift experiments. We observed a large variation in Rubisco carboxylation kinetics among the selected species, although no correlation was found between either the Michaelis–Menten constant for CO_2_ (*K*_c_) or Rubisco content per total soluble protein ([Rubisco]/[TSP]) and the ability to use bicarbonate for non-green seaweeds. This study reports intraspecific Rubisco cold adaptation by means of either higher Rubisco carboxylation turnover rate (*k*_cat_^c^) and carboxylase efficiency (*k*_cat_^c^/*K*_c_) at 4 °C or higher [Rubisco]/[TSP] in some of the analyzed species. Our data point to a widespread ability for photosynthetic bicarbonate usage among polar seaweeds, despite the higher affinity of Rubisco for CO_2_ and higher dissolved CO_2_ concentration in cold seawater. Moreover, the reported catalytic variation within form ID Rubisco might avert the canonical trade-off previously observed between *K*_c_ and *k*_cat_^c^ for plant Rubiscos.

## Teaser

Intraspecific cold-adaptation strategies of Rubisco carboxylation kinetics and content, along with a widespread ability for photosynthetic bicarbonate usage, are reported for polar seaweeds.

## Introduction

Littoral and sublittoral hardbottom zones of polar coastal regions are mainly dominated by dense macroalgal communities, which represent a major trophic contribution to these ecosystems (Dunton and Schell, 1987; Amsler *et al.*, 1995; Iken *et al.*, 1998). The productivity of these communities is comparable to that of temperate seaweed forests (Quartino and Boraso de Zaixso, 2008; Hop *et al.*, 2012), despite the low temperatures in polar regions. Thus, polar seaweeds may have developed photosynthetic adaptations to cold waters, although little is known about the specific molecular processes involved in inorganic carbon (C_i_) acquisition and assimilation in these organisms.

The first major step of photosynthetic carbon fixation is catalyzed by the enzyme ribulose-1,5-bisphosphate carboxylase oxygenase (Rubisco, EC 4.1.1.39). Rubisco is present in most autotrophic organisms from prokaryotes, such as photosynthetic anaerobic bacteria and cyanobacteria, to eukaryotes, such as algae and higher plants (Whitney *et al.*, 2011). Almost all O_2_ evolvers have a form I Rubisco, which consists of eight large and eight small subunits and is subdivided into forms IA–ID depending on its sequence and lineage (Tabita *et al.*, 2008). Plants, green algae, and most cyanobacteria belong to the ‘green’ chloroplast lineage possessing form IB Rubiscos, while non-green algae from the ‘red’ chloroplast lineage contain form ID Rubiscos (Raven and Beardall, 2003; Falkowski *et al.*, 2004).

In spite of their relevance in global carbon cycling, all Rubiscos have a relatively slow carboxylation turnover rate (*k*_cat_^c^) and low affinity for CO_2_, along with poor discrimination between CO_2_ and O_2_ (Spreitzer and Salvucci, 2002). These catalytic traits, together with the low levels of dissolved CO_2_ and slow CO_2_ diffusion rate in water, have led to the evolution of carbon concentrating mechanisms (CCMs) in most aquatic photosynthetic organisms (Giordano *et al.*, 2005). CCMs consist of an active inﬂux of CO_2_ and/or HCO_3_^−^ at the plasmalemma and/or a plastid envelope membrane, which act to increase the CO_2_ concentration around Rubisco (Maberly *et al.*, 1992; Raven and Beardall, 2003; Giordano *et al.*, 2005). Only a few seaweed species appear to lack CCMs (in these species, photosynthesis relies only on passive diffusion of CO_2_), although in many cases stronger evidence on the presence or absence of CCMs is required (Raven *et al.*, 2005).

Different physiological measurements have been widely used to suggest the presence or absence of CCMs; these include the pH compensation point, the activity of transporters or enzymes that are part of a CCM, and the natural ^13^C/^12^C ratio in the algal biomass, among others. The ^13^C isotope discrimination of the algal biomass (δ^13^C_alga_), relative to the isotope composition of the C_i_ source, is primarily controlled by the interplay between C_i_ supply and demand, which affects CO_2_/HCO_3_^−^ acquisition and accumulation (i.e. CCMs), as well as CO_2_ fixation and CO_2_ leakage out of the cell (Sharkey and Berry, 1985). It has been frequently used as a proxy for CCM operation in seaweeds, assuming low leakage, since the increase in the utilization of isotopically heavier HCO_3_^−^ relative to isotopically lighter CO_2_ produces a decrease in the ^13^C isotope discrimination of the algal biomass (higher δ^13^C_alga_) with respect to the isotope fractionation of Rubisco (Raven *et al.*, 2002*a*). The pH compensation point indicates the condition in which the dissolved inorganic carbon (DIC) taken up by the alga equals the CO_2_ released into the medium by respiration and/or photorespiration. Maberly *et al.* (1992) reported δ^13^C_alga_ values more negative than or equal to –30‰ for all seaweeds showing pH compensation points lower than 9.0, that is, the pH at which the dissolved CO_2_ concentration is nearly zero. Since the ratio of the Rubisco reaction rate with ^12^CO_2_ to that with ^13^CO_2_ is 1.030 (at least for spinach Rubisco, Roeske and O’Leary, 1984; see Boller *et al.*, 2015 for other Rubisco types), both measurements suggest that photosynthesis in these seaweeds might rely only on diffusive CO_2_ entry. These species are mainly red algae from the class Florideophyceae inhabiting subtidal habitats, where light is presumed to be the limiting resource (Maberly *et al.*, 1992; Raven *et al.*, 2002*a*).

While the production and operation of CCMs are energy- and resource-demanding processes, diffusive entry of CO_2_ for photosynthesis at the current CO_2_ levels may allow signiﬁcant oxygenase activity of Rubisco, thereby decreasing the efficiency of photosynthesis; in addition, the photorespiratory metabolism also has an associated energetic cost that depends on the Rubisco CO_2_/O_2_ specificity factor (*S*_C/O_; Raven *et al.*, 2014). Thus, Rubisco may have evolved towards an increased affinity for CO_2_, that is, a decreased Michaelis–Menten affinity constant for CO_2_ (*K*_c_; Nisbet *et al.*, 2007) in those organisms relying only on diffusive entry of CO_2_. In fact, in the few rhodophyte species analyzed to date, Rubisco has a high *S*_C/O_ value (Badger *et al.*, 1998). However, most of these rhodophytes are thermo-acidophiles, which are not representative of all species belonging to this phylum. On the other hand, the evolution of CCMs has probably removed some of the pressure to enhance carboxylation efficiency (Meyer and Griffiths, 2013). In this sense, Rubisco from green algae with CCMs and cyanobacteria were found to have high *K*_c_ and *k*_cat_^c^ (Yeoh *et al.*, 1981; Andrews and Lorimer, 1985; Mueller-Cajar and Whitney, 2008). It is still unknown whether the same applies to ID Rubiscos from non-green algae possessing CCMs. Recently, Young *et al.* (2016) have reported that Rubisco from diatoms, which have efficient CCMs, exhibits a broad range of *K*_c_ values that mostly exceed those of C_4_ plant Rubisco, but similar and less variable *k*_cat_^c^ values, suggesting that the canonical trade-off typically observed between *K*_c_ and *k*_cat_^c^ for plant form IB Rubisco might not exist for form ID Rubisco. Nevertheless, more data are required to confirm this observation.

Raven *et al.* (2002*b*) suggested that the impact of low seawater temperatures on photosynthesis would favor diffusive CO_2_ entry rather than CCM operation. The affinity for CO_2_ and *S*_C/O_ of Rubisco increase at decreasing temperatures, while the carboxylation efficiency notably decreases (*k*_cat_^c^/*K*_c_; Jordan and Ogren, 1984; Perdomo *et al.*, 2015; Galmés *et al.*, 2016). It has been hypothesized that the rate of CO_2_ supply to Rubisco would not change significantly at lower temperatures, since the elevated concentrations of dissolved CO_2_ in cold waters mostly compensate for the lower diffusion coefficient of CO_2_ (Raven *et al.*, 2002*b*); so, assuming no increase in Rubisco content at low temperatures, the reduced carboxylation efficiency would lead to a greater CO_2_ concentration around Rubisco (C_c_) during steady-state photosynthesis. However, the previous assumption might be altered by other factors, including changes in the activation state of the enzyme, a probable decrease in the contribution of respiratory and photorespiratory CO_2_, and a larger diffusion boundary layer thickness and changes in transmembrane components affecting the conductance for CO_2_.

In temperate seaweeds, the acclimation of photosynthesis to low temperature involved the production of high concentrations of Calvin cycle enzymes (Davison, 1987). Similarly, higher plants grown at low temperatures up-regulate their Rubisco content, compensating for the intrinsic decline in *k*_cat_^c^ (Holaday *et al.*, 1992; Yamori *et al.*, 2005). This significant increase in Rubisco content might be energetically costly for cold-adapted organisms and requires a high nitrogen (N) investment. Alternatively, photosynthetic adaptation to cold environments by means of higher Rubisco *k*_cat_^c^ values, allowing for a partial compensation of the intrinsic decline in enzymatic activity at low temperatures, would represent N and energy savings for the organism. In this regard, Rubiscos from plants belonging to colder climates typically showed a 40% faster *k*_cat_^c^ than warm-adapted species when both groups were measured at a standard temperature of 25–30 °C (Sage, 2002; Galmés *et al.*, 2015). Unfortunately, Rubisco kinetics have been barely examined in seaweeds, except for a few species (Yeoh *et al.*, 1981; Johnston, 1991; Whitney *et al.*, 2001; Israel and Hophy, 2002); most of these studies measured *K*_c_ at only a single temperature and none of them included polar species. Therefore, more data are needed to explore adaptation patterns in Rubisco investment, kinetics, and C_i_ acquisition mechanisms in seaweeds from different latitudes. This knowledge would be especially useful for the prediction of the consequences of global change in macroalgal communities at high latitudes. The aim of the present study was to identify the presence of adaptive traits in Rubisco kinetics and Rubisco content of macroalgal species representative of Arctic and Antarctic ecosystems, to compare them with populations of the same species from cold-temperate latitudes when possible, and to relate these data to the presence or absence of CCMs in these species.

## Materials and methods

### Algal material

Twelve different macroalgal species representative of Arctic and Antarctic ecosystems, including some populations of the same species from cold-temperate latitudes, were analyzed. The selected species included taxa with contrasting habitat ecologies and evolutionary histories (see Table 1). The analyzed polar and cold-temperate populations belonging to the same species had previously been identified as different ecotypes (Bischoff and Wiencke, 1995; Van de Poll *et al.*, 2002; Olischläger *et al.*, 2017). For some of the species, samples were collected by divers during July 2013 in Kongsfjorden, Spitsbergen, Svalbard (79°N, 11°E), or May 2015 in Helgoland, Germany (54°N, 7°E), and immediately taken to the laboratory in black plastic bags. Young (when possible) and visually healthy specimens free from macroscopic epibiota were chosen for the analyses. For the remainder of the species studied, young macrothalli were raised from the Alfred Wegener Institute (AWI, Bremerhaven, Germany) stock cultures. Cultivation methods were as described by Wiencke and tom Dieck (1989). *Saccharina latissima* from Helgoland was grown at 17±1 °C, and *Palmaria palmata* from Roscoff, *Acrosiphonia arcta* from Helgoland, and *S. latissima* from Spitsbergen were grown at 10±1 °C. These species were cultured using a 16:8 h light:dark photoperiod and a constant photon ﬂuence rate (PFR) of 25–30 µmol photons m^−2^ s^−1^ provided by white light fluorescent tubes (Osram 58W/965 Biolux). The rest of the polar species were grown at 1±1 °C with a changing photoperiod from 5 to 20 h of light to mimic high-latitude seasonal conditions. PFR was measured by means of a quantum ﬂat-head PAR sensor connected to a radiometer (LI-190 and LI-250A, LI-COR Biosciences). Samples for determination of relative Rubisco content, kinetic characterization, and carbon stable isotope composition were snap frozen in liquid nitrogen and stored at –80 °C until analysis.

**Table 1.** Location, origin, evolutionary history, depth zonation, optimum temperature for growth (T_growth_), and upper survival temperature (UST) of the seaweed populations studied

**Species**	**Location of sampling**	**Origin**	**Depth of collection below sea level (m)**	**Evolutionary history**	**Depth zonation**	**T**_**growth**_ (°**C)**	**UST (** °**C)**	**References for T**_**growth**_ **and UST**
**Rhodophyta**								
*Phycodrys rubens* (L.) Batters	Kongsfjord (Spitsbergen)	Natural population	15	Non-endemic	Lower sublittoral	10	n.d.	Novaczek *et al.* (1990)
*Phycodrys rubens* (L.) Batters	Helgoland (Germany)	Natural population	6–8	Non-endemic	Lower sublittoral	n.d.	18–20	Lüning (1984)
*Ptilota gunneri* Silva, Maggs & Irvine	Kongsfjord (Spitsbergen)	Natural population	10	Non-endemic	Lower sublittoral	4–10	n.d.	Gordillo *et al.* (2016)
*Devaleraea ramentacea* (L.) Guiry	Kongsfjord (Spitsbergen)	Natural population	2–3	Arctic Endemic	Upper sublittoral	0–10 (0)	18–20	Novaczek *et al.* (1990); Bischoff and Wiencke (1993)
*Palmaria palmata* (L.) Weber & Mohr	Kongsfjord (Spitsbergen)	AWI collection (2265)	–	Non-endemic	Upper sublittoral	12	n.d.	Van de Poll *et al.* (2002)
*Palmaria palmata* (L.) Weber & Mohr	Roscoff (France)	AWI collection	–	Non-endemic	Lower eulittoral/upper sublittoral	12	n.d.	Van de Poll *et al.* (2002)
*Palmaria decipiens* (Reinsch) Ricker	King George Island (Antarctica)	AWI collection (2113)	–	Antarctic Endemic	Mid eulittoral/upper sublittoral	5	15–16	Wiencke and tom Dieck (1989)
**Ochrophyta (Phaeophyceae)**								
*Alaria esculenta* (L.) Greville	Kongsfjord (Spitsbergen)	Natural population	10	Non-endemic	Mid sublittoral	4–10	n.d.	Gordillo *et al.* (2016)
*Desmarestia aculeata* (L.) Lamouroux	Kongsfjord (Spitsbergen)	Natural population	5	Non-endemic	Mid sublittoral	5	20	Bischoff and Wiencke (1993)
*Laminaria solidungula* J.Agardh	Kongsfjord (Spitsbergen)	Natural population	4–6	Arctic Endemic	Mid sublittoral	5–10	16	tom Dieck (1992)
*Laminaria digitata* (Huds.) Lamouroux	Kongsfjord (Spitsbergen)	Natural population	5	Non-endemic	Mid sublittoral	10	n.d.	Bolton and Lüning (1982)
*Saccharina latissima* (L.) Lane, Mayes, Druehl, Saunders	Kongsfjord (Spitsbergen)	AWI collection(3123,3124)	–	Non-endemic	Mid sublittoral	10	n.d.	Olischläger *et al.* (2017)
*Saccharina latissima* (L.) Lane, Mayes, Druehl, Saunders	Helgoland (Germany)	AWI collection(3094,3096)	–	Non-endemic	Mid sublittoral	10–20 (15)	18–20	Fortes and Lüning (1980)
*Himantothallus grandifolius* (A. & E.S.Gepp) Zinova	King George Island (Antarctica)	AWI collection(3006,3010)	–	Antarctic Endemic	Mid to lower sublittoral	0–5	11–13	Wiencke and tom Dieck (1989)
**Chlorophyta**								
*Acrosiphonia arcta* (Dillwyn) Gain	Disko Island (Greenland)	AWI collection(1120)	–	Non-endemic	Upper sublittoral	0–10	22	Bischoff and Wiencke (1995)
*Acrosiphonia arcta* (Dillwyn) Gain	Helgoland (Germany)	AWI collection(1083)	–	Non-endemic	Lower eulittoral	5–15	22	Bischoff and Wiencke (1995)
*Acrosiphonia arcta* (Dillwyn) Gain	King George Island (Antarctica)	AWI collection(1160)	–	Non-endemic	Lower eulittoral	5	22	Bischoff and Wiencke (1995)

n.d., Not determined.

### Carbon stable isotope composition

The abundance of ^13^C relative to ^12^C in the algal samples was determined by mass spectrometry using a DELTA V Advantage (Thermo Electron Corporation) isotope ratio mass spectrometer (IRMS) connected to a Flash EA 1112 CNH analyzer (Thermo Electron Corporation). The calculated δ^13^C_alga_ was corrected with the isotope composition of DIC found in the medium from which the samples were collected (δ^13^C_DIC_), as described by Iñiguez *et al.* (2016*a*, *b*). Measurements of δ^13^C_DIC_ were done with the same IRMS connected to a GasBench II system (Thermo Electron Corporation).

### pH drift

Specimens were placed in 100 ml glass bottles completely filled with 0.2 µm-ﬁltered seawater, with magnetic stirring, and tightly sealed to avoid gas exchange with the air. Assays were performed at saturating PFR (previously determined for each species by chlorophyll *a* fluorescence rapid light curves by means of a pulse-amplitude-modulated fluorometer; Mini-PAM, Walz) at their respective growth temperatures for specimens from stock cultures, or at 3±1 °C for the species collected in Kongsfjorden. Specimen size was adjusted to prevent self-shading and to ensure effective agitation of the medium. The pH was recorded using a thin glass electrode (CRISON 52 09, pH meter CRISON GLP 22) until it reached a stable reading (after ~24 hours of incubation), which represents the pH compensation point.

### Rubisco content and carboxylase kinetics

Rubisco carboxylation kinetics (*k*_cat_^c^ and *K*_c_) were determined at 25 °C and 4 °C in rapid crude protein extracts, according to the methods described for plants (Sharwood *et al.*, 2008, 2016) and algae (Heureux *et al.*, 2017; Young *et al.*, 2016). Rapidly prepared fresh extracts instead of purified Rubisco preparations were used to prevent the degradation of Rubisco because the C-terminal loop of the enzyme is often a target for proteases, which results in changes in the catalytic performance (Sharwood *et al.*, 2008). Approximately 0.5 g fresh weight of frozen samples were homogenized in a pre-chilled Mixer Mill (MM 400, Retsch) with 1 ml of ice-cold extraction buffer consisting of 100 mM Bicine-NaOH CO_2_-free (pH 8.1), 1 mM EDTA, 10 mM DTT, 50 mM β-mercaptoethanol, 20 mM MgCl_2_, 1 mM benzamidine, 1 mM ε-aminocaproic acid, 1% plant protease inhibitor cocktail (P9599, Sigma-Aldrich), 2 mM phenylmethylsulfonyl ﬂuoride, 0.5% Triton X-100, 25 mg ml^−1^ polyvinylpolypyrrolidone, and a saturating concentration of NaHCO_3_ (10 mM, 20 mM, or 40 mM depending on the species). The homogenate was immediately centrifuged for 10 min at 20,000 *g* and 4 °C. Total soluble protein was determined in the supernatant according to the method of Bradford (1976). Part of the supernatant was then supplemented with sufficient carrier-free NaH^14^CO_3_ to adjust the specific radioactivity to 3.7 × 10^10^ Bq mol^−1^ (1 Ci mol^−1^) and incubated for 15–20 min at 25 °C for full activation of Rubisco. Rates of Rubisco ^14^CO_2_ fixation were measured in 8 ml septum-sealed glass vials with magnetic stirring, under a 100% N_2_ atmosphere. The vials contained assay buffer, consisting of 100 mM Bicine-NaOH CO_2_-free (pH 8.1), 20 mM MgCl_2_, 1.5 mM ribulose-1,5-bisphosphate (RuBP; R0878, Sigma-Aldrich) and ~100 W-A units of carbonic anhydrase (C3934, Sigma-Aldrich), previously sparged with 100% N_2_, and one of eight concentrations of NaH^14^CO_3_ from 0.2 to 18 mM for the assays at 25 °C and from 0.2 to 6 mM for the assays at 4 °C (except for the green algae, for which NaH^14^CO_3_ concentrations from 0.8 to 49 mM for the assays at 25 °C and from 0.8 to 17 mM for the assays at 4 °C were used), each with a specific radioactivity of 3.7 × 10^10^ Bq mol^−1^. Assays (0.5 ml final volume) were started by the injection of 10–20 µl of activated algal extract and stopped with the addition of 200 µl 1 M formic acid after 1 min (for the assays at 25 °C) or 2 min (for the assays at 4 °C). The acidiﬁed samples were dried at 80 °C and the acid-stable ^14^C-organic molecules were determined by scintillation counting (Beckman Coulter LS 6500). Values for *K*_c_ and maximum carboxylase activity (*V*_max_^c^) were extrapolated from the data fitted to the Michaelis–Menten equation as described by Sharwood *et al.* (2008) and Whitney *et al.* (2011). Concentrations of CO_2_ in solution were calculated assuming an acid dissociation constant (p*K*_a_) for carbonic acid of 6.28 at 4 °C and 6.11 at 25 °C (Galmés *et al.*, 2016), a solubility constant for CO_2_ of 0.0626 mol l^−1^ atm^−1^ at 4 °C and 0.034 mol l^−1^ atm^−1^ at 25 °C, and using accurate measures of the pH (NBS scale) of each buffer solution at the respective assay temperature. Replicate measurements (*n*=3–5) were made using independent crude protein extracts from different individuals. A series of assays of *Triticum aestivum* L. cv. Cajeme was interspersed with those of the algal species analyzed as an external control, yielding values similar to those recently reported in the literature (Hermida-Carrera *et al.*, 2016; Table 2).

**Table 2.** Rubisco carboxylation kinetics of the analyzed polar populations measured at 25 °C and 4 °C, the ratio between 25 °C and 4 °C measurements for each kinetic parameter, and Rubisco content at the growth conditions

**Species**	***K***_**c**_ **(µM)**			***k***_**cat**_^**c**^ **(s** ^−**1**^)			***k***_**cat**_^**c**^ **/*K***_**c**_ **(s** ^−**1**^ **mM** ^−**1**^)			**[Rubisco]/** **[TSP] (%)**
	**25** °**C**	**4** °**C**	**25** °**C/4** °**C**	**25** °**C**	**4** °**C**	**25** °**C/4** °**C**	**25** °**C**	**4** °**C**	**25** °**C/4** °**C**	
**Rhodophyta**										
* Phycodrys rubens*	18.9±0.8	4.8±0.7	3.94±0.40	1.76±0.03	0.14±0.01	12.2±0.9	93.5±3.8	30.1±2.0	3.11±0.10	8.0±0.7
* Ptilota gunneri*	14.4±1.1	5.1±0.6	2.83±0.12	1.60±0.23	0.17±0.03	9.3±0.5	110.7±7.2	33.8±2.4	3.27±0.05	6.6±0.9
* Devaleraea ramentacea*	17.5±1.0	5.6±0.8	3.13±0.26	2.59±0.16	0.34±0.04	7.6±0.6	148.1±8.3	60.9±0.9	2.43±0.14	7.4±1.7
* Palmaria palmata*	15.9±0.6	4.9±0.2	3.24±0.01	2.08±0.05	0.27±0.01	7.8±0.2	131.3±8.1	54.6±2.1	2.40±0.07	9.9±0.6
* Palmaria decipiens*	17.4±1.2	5.0±0.2	3.50±0.12	2.43±0.16	0.31±0.01	7.8±0.2	139.7±3.3	62.9±0.3	2.22±0.05	7.8±0.1
**Ochrophyta (Phaeophyceae)**										
* Alaria esculenta*	23.6±1.0	4.1±0.5	5.85±1.01	2.13±0.14	0.23±0.02	9.5±1.3	90.3±4.3	55.5±1.9	1.63±0.06	17.4±1.8
* Desmarestia aculeata*	13.3±0.6	2.1±0.3	6.44±0.89	1.37±0.12	0.12±0.02	11.3 ±1.3	103.3±12	61.0±22	1.79±0.41	17.7±4.7
* Laminaria solidungula*	18.5±1.3	3.9±0.8	4.96±1.23	1.60±0.12	0.17±0.02	9.2±0.7	86.6±6.2	47.1±14	1.92±0.41	30.1±1.4
* Laminaria digitata*	17.0±1.0	4.0±0.3	4.25±0.40	1.42±0.28	0.10±0.02	16.4±2.9	84.3±20	26.1±2.8	3.84±0.36	24.7±7.5
* Saccharina latissima*	19.4±1.2	3.8±0.4	4.99±0.29	1.79±0.18	0.16±0.03	10.8±0.6	92.5±7.5	43.3±8.4	2.17±0.23	37.3±6.1
* Himantothallus grandifolius*	18.1±1.1	4.4±0.8	4.14±0.49	2.08±0.02	0.23±0.01	9.2±0.6	115±7.4	51.7±6.5	2.23±0.16	n.d.
**Chlorophyta**										
* Acrosiphonia arcta* (Arctic)	52.8±1.6	17.7±2.6	3.01±0.33	5.08±0.15	0.82±0.03	6.2±0.1	96.2±2.7	46.7±5.7	2.07±0.22	7.6±0.1
**Control**										
* Triticum aestivum*	9.6±0.4	3.1±0.1	3.06±0.14	2.20±0.23	0.20±0.01	11.2±0.6	230.1±28	62.8±4.1	3.66±0.28	42.3±2.5

***k***_**cat**_^**c**^, Rubisco carboxylase turnover rate; *K*_c_, Michaelis–Menten affinity constant for CO_2_; ***k***_**cat**_^**c**^**/***K*_**c**_, carboxylation efficiency; [Rubisco]/[TSP], percentage of Rubisco in the total soluble protein. Data are means ±SD (*n*=3–5 independent thalli). n.d., Not determined.

*k*_cat_^c^ was calculated by dividing *V*_max_ by the concentration of Rubisco active sites, which was quantified from the same crude protein extracts by 2ʹ-carboxyarabinitol-1,5-bisphosphate (^14^C-CABP) binding (Ruuska *et al.*, 1998), assuming eight binding sites per Rubisco (Blayney *et al.*, 2011). Our preliminary assays revealed that analyzed red and brown seaweed Rubiscos need an increased CABP concentration (up to 1.2 mM) for saturating Rubisco active sites than that previously used for form IB Rubiscos (29–80 µM; Ruuska *et al.*, 1998; Kubien *et al.*, 2011). These results agree well with those from Pearce (2006) showing a 100-fold higher semi-saturation constant for CABP inhibition and 2-fold slower binding of form ID than form IB Rubisco active sites. A 15 µl aliquot of activated extract was incubated with ^14^C-CABP for 30 min at room temperature before chromatographic separation of Rubisco-bound and free ^14^C-CABP. Previous incubation of the mixture for up to 24 h at 4 °C followed by 30 min at room temperature before chromatographic separation did not significantly increase ^14^C-CABP binding. Immunoblotting of the crude protein extracts using a Rubisco large subunit antibody and purified spinach Rubisco standard (AS03 037 and AS01 017S, Agrisera) gave similar values of Rubisco concentration to those obtained by ^14^C-CABP binding (data not shown).

Carboxylation assay controls at saturating NaH^14^CO_3_ concentrations either without RuBP addition or with activated algal extract pre-incubated for 30 min with non-radioactive CABP (up to 1.2 mM) were carried out in order to confirm that the observed acid-stable ^14^C was only the result of Rubisco catalytic activity. Both controls gave values lower than 5% of the maximum activity in all analyzed species. Saturating concentrations of RuBP, ^14^C-CABP, and H^14^CO_3_^−^, as well as the optimum incubation time and temperature for full Rubisco activation, were determined in preliminary assays. RuBP of ≥90% purity (R0878, Sigma-Aldrich) and of ≥99% purity (83895, Sigma-Aldrich) were also compared, obtaining no significant differences in Rubisco kinetics at saturating conditions.

### Data analysis

Significance of differences between populations of the same species (*n*=3–5) were tested using one-way analysis of variance, after normality (Shapiro–Wilk test) and homogeneity of variances were confirmed. Post hoc comparisons were performed using Fisher’s least significant difference test. Pearson correlation coefficients were obtained for the significance of the association between different variables. The confidence interval for all these tests was set at 95% (*P*≤0.05). All statistical analyses were performed using SigmaPlot 12.0 statistical software (Systat Software Inc.).

## Results

### Variability in Rubisco kinetics and its thermal response among polar seaweeds

At 25 °C, when considering only form ID Rubiscos (i.e. rhodophytes and ochrophytes), *K*_c_ and *k*_cat_^c^ varied ~2-fold among species (Table 2). The lowest values corresponded to *Desmarestia aculeata* (*K*_c_=13.3 μM, *k*_cat_^c^=1.37 s^−1^) and the highest values were found in *Alaria esculenta* for *K*_c_ (23.6 µM) and in *Devaleraea ramentacea* for *k*_cat_^c^ (2.59 s^−1^). Rubisco from *A. arcta,* the only chlorophyte included in the study, had the highest values for *K*_c_ and *k*_cat_^c^, which were two to four times higher than those found in the other species.

The highest values of *k*_cat_^c^/*K*_c_ were found for the polar endemic species *Palmaria decipiens* (140 s^−1^ mM^−1^) and *D. ramentacea* (148 s^−1^ mM^−1^), while the lowest value was obtained for *Laminaria digitata* (84.3 s^−1^ mM^−1^; Table 2). The Antarctic endemic species *Himantothallus grandifolius* presented the highest value among the ochrophytes (115 s^−1^ mM^−1^). The chlorophyte *A. arcta* had a *k*_cat_^c^/*K*_c_ in the range of the other species.

The percentage of Rubisco in the total soluble protein ([Rubisco]/[TSP]) was highly variable among the species, ranging from 6.6% in *Ptilota gunneri* to 37.3% in *S. latissima* (Table 2).

The range of variation for *K*_c_ and *k*_cat_^c^ was smaller at 4 °C compared with 25 °C (Table 2). Among form ID Rubiscos, at 4 °C, *K*_c_ and *k*_cat_^c^ ranged from 2.1 µM (*D. aculeata*) and 0.1 s^−1^ (*L. digitata*) to 5.6 µM and 0.34 s^−1^ (both for *D. ramentacea*), respectively. The chlorophyte *A. arcta* again showed the highest values for *K*_c_ and *k*_cat_^c^ at 4 °C, with values being three to eight times higher than those in the remaining species. At 4 °C, the species showing maximum and minimum values of *k*_cat_^c^/*K*_c_ were the same as those identified at 25 °C, with the exception of *D. aculeata*, which presented one of the highest values (61 s^−1^ mM^−1^).

Regarding thermal dependencies of the different kinetics parameters, the ratio (*K*_c_)^25 °C^/(*K*_c_)^4 °C^ varied between 2.83 for *Ptilota gunneri* and 6.44 for *D. aculeata*, while (*k*_cat_^c^)^25 °C^/(*k*_cat_^c^)^4 °C^ ranged from 6.2 in *A. arcta* to 16.4 in *L. digitata* (Table 2). The lowest value of (*k*_cat_^c^)^25 °C^/(*k*_cat_^c^)^4 °C^ within the Rhodophyta was obtained for the Arctic endemic species *D. ramentacea* (7.6), and within the Ochrophyta, for the two polar endemic species *Laminaria solidungula* and *H. grandifolius* (9.2), whereas (*k*_cat_^c^/*K*_c_)^25 °C^/(*k*_cat_^c^/*K*_c_)^4 °C^ varied from 1.63 in *A. esculenta* to 3.84 in *L. digitata*.

### Intraspecific differences in Rubisco kinetics and its thermal response between polar and cold-temperate populations

For *Phycodrys rubens*, the only statistically significant difference between the Arctic and cold-temperate populations was found in *K*_c_ and *k*_cat_^c^/*K*_c_ at 25 °C, consisting of a lower affinity for CO_2_ and a lower carboxylation efficiency in the polar population compared with the Atlantic population (Table 3). These differences at 25 °C were not found at 4 °C, and, furthermore, [Rubisco]/[TSP], *k*_cat_^c^, and the ratio 25 °C/4 °C for the different kinetic parameters were not significantly different between the two populations.

**Table 3.** Rubisco carboxylation kinetics of the polar and cold-temperate populations of the same species measured at 25 °C and 4ºC, the ratio between 25 °C and 4 °C measurements for each kinetic parameter, and Rubisco content at the growth conditions (data corresponding to the polar populations are also shown in Table 2)

**Species**	***K***_**c**_ **(µM)**			***k***_**cat**_^**c**^ **(s** ^−**1**^)			***k***_**cat**_^**c**^ **/*K***_**c**_ **(s** ^−**1**^ **mM** ^−**1**^)			**[Rubisco]/** **[TSP] (%)**
	**25** °**C**	**4** °**C**	**25** °**C/4** °**C**	**25** °**C**	**4** °**C**	**25** °**C/4** °**C**	**25** °**C**	**4** °**C**	**25** °**C/4** °**C**	
**Rhodophyta**										
* Phycodrys rubens* (Arctic)	18.9±0.8 ^b^	4.8±0.7 ^a^	3.94±0.40 ^a^	1.76±0.03 ^a^	0.14±0.01 ^a^	12.2±0.9 ^a^	93.5±3.8 ^a^	30.1±2.0 ^a^	3.11±0.10 ^a^	8.0±0.7 ^a^
* Phycodrys rubens* (Helgoland)	15.7±0.7 ^a^	5.3±1.9 ^a^	3.24±1.14 ^a^	1.92±0.19 ^a^	0.16±0.03 ^a^	12.1±1.3 ^a^	122±10.6 ^b^	31.9±6.8 ^a^	3.95±0.90 ^a^	8.5±0.3 ^a^
* Palmaria palmata* (Arctic)	15.9±0.6 ^a^	4.9±0.2 ^a^	3.24±0.01 ^a^	2.08±0.05 ^b^	0.27±0.01 ^b^	7.8±0.2 ^a^	131.3±8.1 ^a^	54.6±2.1 ^b^	2.40±0.07 ^a^	9.9±0.6 ^a^
* Palmaria palmata* (Roscoff)	14.9±0.8 ^a^	4.6±0.1 ^a^	3.22±0.22 ^a^	1.85±0.04 ^a^	0.23±0.01 ^a^	8.2±0.3 ^a^	124.8±9.4 ^a^	49.1±2.6 ^a^	2.54±0.09 ^a^	9.4±0.2 ^a^
**Ochrophyta (Phaeophyceae)**										
* Saccharina latissima* (Arctic)	19.4±1.2 ^a^	3.8±0.4 ^a^	4.99±0.29 ^a^	1.79±0.18 ^a^	0.16±0.03 ^b^	10.8±0.6 ^a^	92.5±7.5 ^b^	43.3±8.4 ^b^	2.17±0.23 ^a^	37.3±6.1 ^a^
* Saccharina latissima* (Helgoland)	19.6±0.5 ^a^	4.2±0.6 ^a^	4.75±0.61 ^a^	1.34±0.44 ^a^	0.11±0.04 ^a^	12.6±0.7 ^b^	68.1±21.5 ^a^	26.3±10 ^a^	2.69±0.38 ^b^	36.6±6.4 ^a^
**Chlorophyta**										
* Acrosiphonia arcta* (Arctic)	52.8±1.6 ^b^	17.7±2.6 ^a^	3.01±0.33 ^a^	5.08±0.15 ^a^	0.82±0.03 ^a^	6.19±0.07 ^c^	96.2±2.7 ^b^	46.7±5.7 ^a^	2.07±0.22 ^b^	7.6±0.1 ^b^
* Acrosiphonia arcta* (Helgoland)	57.4±1.6 ^c^	16.4±0.6 ^a^	3.49±0.16 ^a^	4.89±0.06 ^a^	0.82±0.02 ^a^	5.94±0.11 ^b^	85.3±2.9 ^a^	50.1±0.9 ^a^	1.70±0.09 ^a^	2.9±0.4 ^a^
* Acrosiphonia arcta* (Antarctic)	48.2±0.9 ^a^	15.6±1.5 ^a^	3.10±0.25 ^a^	4.99±0.02 ^a^	0.86±0.01 ^b^	5.78±0.06 ^a^	103.5±2.0 ^c^	55.0±6.0 ^a^	1.87±0.16 ^ab^	6.9±1.4 ^b^

***k***_**cat**_^**c**^, Rubisco carboxylase turnover rate; ***K***_**c**_, Michaelis–Menten affinity constant for CO_**2**_; ***k***_**cat**_^**c**^/*K*_c_, **carboxylation efficiency;** [Rubisco]/[TSP], percentage of Rubisco in the total soluble protein. Different letters indicate statistically significant differences (*P*<0.05) between populations of the same species. Data are means ±SD (*n*=3–5 independent thalli).

For *Palmaria palmata*, the Arctic population displayed ~15% higher *k*_cat_^c^ and *k*_cat_^c^/*K*_c_ at 4 °C compared with the Atlantic population (Table 3). Non-significant differences were observed between the two populations in *K*_c_, [Rubisco]/[TSP], and the ratio 25 °C/4 °C for the different kinetic parameters.

The Arctic population of *S. latissima* had a higher *k*_cat_^c^ at 4 °C compared with the cold-temperate population, together with a higher *k*_cat_^c^/*K*_c_ at both assayed temperatures (65% higher at 4 °C; see Table 3). As a consequence, the ratios (*k*_cat_^c^)^25 °C^/(*k*_cat_^c^)^4 °C^ and (*k*_cat_^c^/*K*_c_)^25 °C^/(*k*_cat_^c^/*K*_c_)^4 °C^ were lower in polar *S. latissima* relative to the Atlantic population, while [Rubisco]/[TSP] was similar in the two populations.

The Antarctic population of *A. arcta* showed the lowest *K*_c_ at 25 °C, followed by the Arctic population, in comparison to the cold-temperate population; this difference was reflected in a higher *k*_cat_^c^/*K*_c_ for the Antarctic population (Table 3). At 4 °C, non-significant differences in *k*_cat_^c^/*K*_c_ were found among the three populations, despite the higher *k*_cat_^c^ for the Antarctic population. Most noticeably, [Rubisco]/[TSP] was ~150% higher in the two polar populations compared with the cold-temperate population.

### Carbon utilization of polar versus cold-temperate populations

**Fig. 1.**
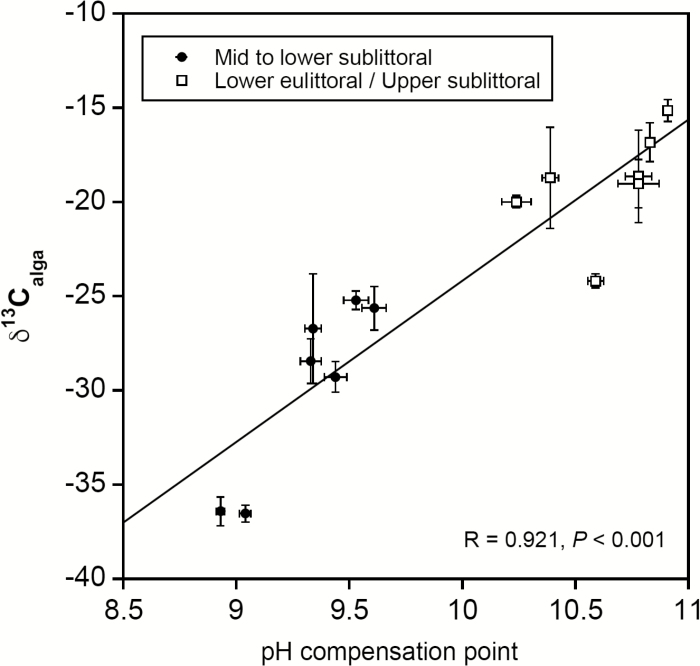
Relationship between the pH compensation point and carbon isotope discrimination (δ^13^C_alga_) from all analyzed seaweed populations. Filled circles represent populations distributed in the mid to lower sublittoral and open squares represent populations distributed in the lower sublittoral and/or upper sublittoral. Data are presented as mean ±SD (*n*=4–5).

Among all the species, only two of them (the lower sublittoral rhodophytes *Phycodrys rubens* and *Ptilota gunneri*) displayed values of δ^13^C_alga_ <–30‰ and pH compensation point ≤9 (Table 4). A positive correlation was found between δ^13^C_alga_ and pH compensation point among all seaweed species studied (*R*=0.921, *P*<0.001, Fig. 1) and also when considering species with form ID Rubisco exclusively (*R*=0.891, *P*<0.001). As shown in Fig. 1, there was a striking separation into two groups, one for the species growing in the mid to lower sublittoral, with δ^13^C_alga_ <–25‰ and pH compensation point <10, and the other for the species growing in the upper sublittoral to lower eulittoral, with δ^13^C_alga_ >–25‰ and pH compensation point >10.

**Table 4.** Stable carbon isotope discrimination values (δ^13^C_alga_) and pH compensation points of the analyzed seaweed populations

**Species**	δ^**13**^**C**_**alga**_**(‰)**	**pH compensation point**
**Rhodophyta**		
*Phycodrys rubens* (Arctic)	–36.5±0.4 ^a^	9.04±0.03
*Phycodrys rubens* (Helgoland)	–37.4±0.5 ^b^	n.d.
*Ptilota gunneri*	–36.4±0.8	8.93±0.02
*Devaleraea ramentacea*	–24.2±0.4	10.59±0.04
*Palmaria palmata* (Arctic)	–18.6±2.4 ^a^	10.78±0.06 ^b^
*Palmaria palmata* (Roscoff)	–18.7±2.7 ^a^	10.39±0.04 ^a^
*Palmaria decipiens*	–19±1.3	10.78±0.09
**Ochrophyta (Phaeophyceae)**		
*Alaria esculenta*	–28.4±1.2	9.33±0.05
*Desmarestia aculeata*	–26.7±2.9	9.34±0.04
*Laminaria solidungula*	–29.3±0.8	9.44±0.05
*Laminaria digitata*	–25.6±1.2	9.61±0.05
*Saccharina latissima* (Arctic)	–21.8±0.8 ^a^	n.d.
*Saccharina latissima* (Helgoland)	–23.7±1.4 ^b^	n.d.
*Himantothallus grandifolius*	–25.2±0.5	9.53±0.06
**Chlorophyta**		
*Acrosiphonia arcta* (Arctic)	–15.1±0.6 ^a^	10.91±0.01 ^b^
*Acrosiphonia arcta* (Helgoland)	–20±0.3 ^c^	10.37±0.03 ^a^
*Acrosiphonia arcta* (Antarctic)	–16.8±1 ^b^	10.83±0.01 ^b^

Different letters indicate statistically significant differences (*P*<0.05) between populations of the same species. Data are means ±SD (*n*=4–5 independent thalli). n.d., Not determined.

All polar populations showed a significantly higher pH compensation point and/or less negative δ^13^C_alga_ values than their cold-temperate counterparts (Table 4). The Atlantic population of *Phycodrys rubens* had a slightly more negative δ^13^C_alga_ value than its polar counterpart, although both values were lower than –30‰. The pH compensation point found in the *Palmaria palmata* population from Roscoff was lower than that from the polar population of the same species, whereas the δ^13^C_alga_ values were similar. For *S. latissima,* the δ^13^C_alga_ value was lower in the Helgoland population relative to the polar population. The Atlantic *A. arcta* showed lower δ^13^C_alga_ and pH compensation point values than the Arctic and Antarctic populations of this species.

### Trade-off among Rubisco carboxylation kinetics and their relationship with carbon utilization

**Fig. 2.**
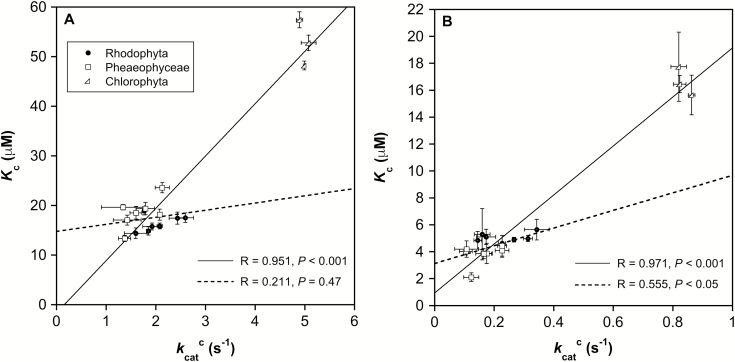
Trade-off between the maximum carboxylation rate (*k*_cat_^c^) and the Michaelis–Menten affinity constant for CO_2_ (*K*_c_) for the analyzed seaweed Rubiscos at (A) 25 °C and (B) 4 ° C. The solid line represents the correlation of all populations together (including chlorophytes); the dashed line represents the correlation of form ID Rubiscos alone. Data are presented as mean ±SD (*n*=3–5).

When analyzing the data from all species assayed at both 4 °C and 25 °C, *k*_cat_^c^ and *K*_*c*_ correlated positively (*R*=0.958, *P*<0.001). The same correlation was also found when considering the species with form ID Rubisco exclusively (*R*=0.935, *P*<0.001). At each assay temperature, the correlation between *k*_cat_^c^ and *K*_c_ was significant when all species were analyzed together (*R*=0.951, *P*<0.001 at 25 °C and *R*=0.971, *P*<0.001 at 4 °C; Fig. 2). However, the analysis of form ID Rubiscos alone revealed a significant correlation only between *k*_cat_^c^ and *K*_c_ at 4 °C (*R*=0.555, *P*<0.05), and not at 25 °C (*R*=0.211, *P*=0.47, Fig. 2).

Generally, a larger number of significant correlations was obtained between Rubisco kinetics and either δ^13^C_alga_ values, pH compensation points, or the ratio [Rubisco]/[TSP] when all species were analyzed together than when the three populations of the chlorophyte *A. arcta* were excluded from the analysis (Table 5). [Rubisco]/[TSP] correlated negatively with *k*_cat_^c^/*K*_c_ at 25 °C. The significant negative correlation between [Rubisco]/[TSP] and *k*_cat_^c^ at both 4 °C and 25 °C was lost when only form ID Rubiscos were analyzed. A positive relationship was found between *k*_cat_^c^ measured at both temperatures and the pH compensation point. The ratio *k*_cat_^c^/*K*_c_ at 25 °C correlated positively with the pH compensation point only for form ID Rubiscos. Conversely, all significant correlations between δ^13^C_alga_ and Rubisco kinetics were lost when only form ID Rubiscos were analyzed (Table 5).

**Table 5.** Pearson’s correlation coefficients between the Rubisco kinetic parameters at 25 °C and 4 °C and either the percentage of Rubisco in the total soluble protein, ^13^C isotope discrimination, or pH compensation point, considering (A) all 17 seaweed populations together, and (B) the 14 red and brown seaweed populations (all possessing form ID Rubiscos) alone

**(A) Data from all populations analyzed together**						
	**25** °**C**			**4** °**C**		
	***K***_**c**_	***k***_**cat**_^**c**^	***k***_**cat**_^**c**^ **/*K***_**c**_	***K***_**c**_	***k***_**cat**_^**c**^	***k***_**cat**_^**c**^ **/*K***_**c**_
**δ** ^**13**^ **C**_**alga**_	0.505*	0.57*	0.1	0.502*	0.601*	0.514*
**pH compensation point**	0.455	0.637*	0.488	0.554*	0.65*	0.519
**[Rubisco]/** **[TSP]**	–0.34	–0.519*	–0.575*	–0.476	–0.513*	–0.339
**(B) Data from red and brown algal species (Form ID Rubiscos)**						
	**25** °**C**			**4** °**C**		
	***K***_**c**_	***k***_**cat**_^**c**^	***k***_**cat**_^**c**^ **/*K***_**c**_	***K***_**c**_	***k***_**cat**_^**c**^	***k***_**cat**_^**c**^ **/*K***_**c**_
**δ** ^**13**^ **C**_**alga**_	0.01	0.27	0.26	0.13	0.44	0.518
**pH compensation point**	–0.15	0.669*	0.806**	0.41	0.777**	0.573
**[Rubisco]/** **[TSP]**	0.42	–0.545	–0.768**	–0.549	–0.536	–0.29

*k*_cat_^c^/*K*_c_, carboxylation efficiency; *k*_cat_^c^, carboxylase turnover rate; *K*_c_, Michaelis–Menten affinity constant for CO_2_; [Rubisco]/[TSP], percentage of Rubisco in the total soluble protein; δ^13^C_alga_, ^13^C isotope discrimination. *P<0.05, **P<0.01, ***P<0.001.

## Discussion

### Variability in Rubisco carboxylation kinetics

**Fig. 3.**
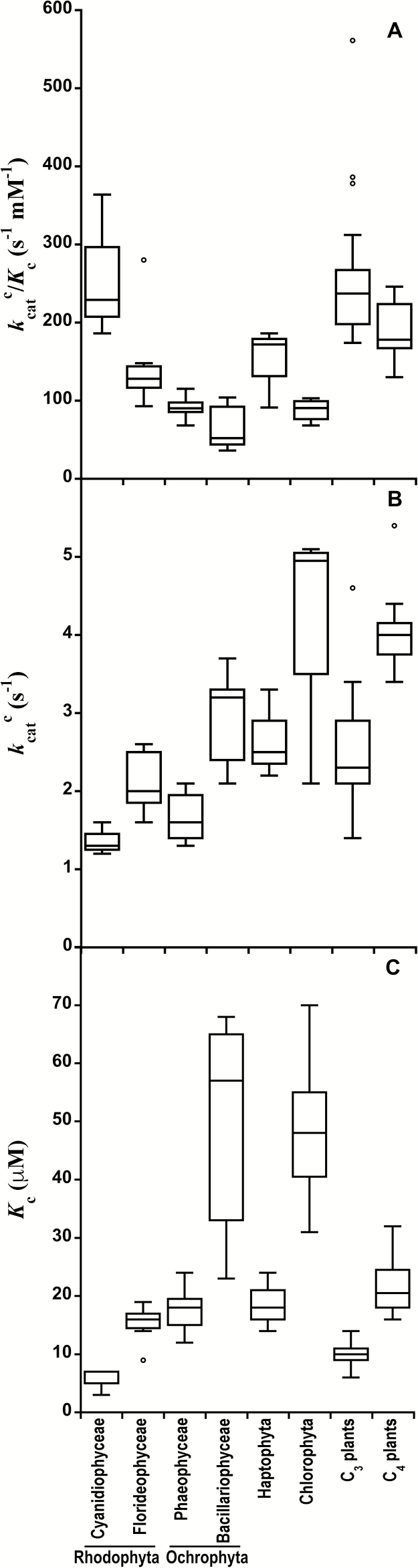
Box plots depicting Rubisco carboxylation kinetics parameters at 25 °C for different taxonomic groups, including the species analyzed in this study (see Tables 2 and 4) and previous published values for others species (see Supplementary Table S1). (A) Carboxylation efficiency (*k*_cat_^c^/*K*_c_); (B) carboxylase turnover rate (*k*_cat_^c^); (C) Michaelis–Menten affinity constant for CO_2_ (*K*_c_). For each plot, the horizontal line represents the median, the box and whiskers represent the 25th to 75th percentile and the minimum to maximum distributions of the data, respectively, and any value outside this range is displayed as an individual point.

The *K*_c_ values obtained in the present study at 25 °C (Tables 2 and 3) fell within the wide range reported for the few seaweed species analyzed in previous studies, between 9.3 µM in *Griffithsia monilis* (Whitney *et al.*, 2001) and 70 µM in *Ulva* sp. (Yeoh *et al.*, 1981). Fig. 3 represents a compilation of our results and previously published carboxylation kinetics measured at 25 °C for other phylogenetic groups possessing form I Rubisco (see Supplementary Table S1 at *JXB* online), showing that brown seaweeds (Phaeophyceae) display lower *K*_c_ and *k*_cat_^c^ values at 25 °C than diatoms (Bacillariophyceae), and lower *k*_cat_^c^ but similar *K*_c_ to haptophytes (which also possess form ID Rubisco). On the other hand, red seaweeds (Florideophyceae) present higher *K*_c_ and *k*_cat_^c^ values and lower *k*_cat_^c^/*K*_c_ than red algae belonging to the family Cyanidiophyceae. These differences reveal a large variation within form ID Rubisco kinetics.

Rubisco *K*_c_ values from brown and red seaweeds, along with those from haptophytes, were similar to those of C_4_ plants and higher than those of C_3_ plants, whereas chlorophytes and diatoms displayed the highest *K*_c_ values within the eukaryotes (Fig. 3C). These differences in *K*_c_ might be related to the presence and strength of CCMs. However, the same differences were not observed in *k*_cat_^c^ values (Fig. 3B), leading to lower carboxylation efficiencies (*k*_cat_^c^/*K*_c_) in all seaweeds analyzed in the present study relative to C_3_ vascular plants; this seems to be a general pattern within marine algae (Fig. 3A). Moreover, Ochrophyta and Chlorophyta also showed significantly lower *k*_cat_^c^/*K*_c_ than C_4_ vascular plants.

The absence of a significant correlation between *K*_c_ and *k*_cat_^c^ at 25 °C when only red and brown seaweed Rubiscos were analyzed is in agreement with the results of Young *et al.* (2016) and Heureux *et al.* (2017) for other form ID Rubiscos. These results suggest that the canonical trade-off typically observed between *K*_c_ and *k*_cat_^c^ for plants, which was thought to be due to a fundamental mechanistic constraint of their interrelated rate constants (Tcherkez *et al.*, 2006), might not be universal for all Rubiscos. Differences in the relationship between *k*_cat_^c^ and *K*_c_ may arise from differences in the intrinsic equilibrium of the RuBP enolization reaction (Tcherkez, 2013).

### Co-evolution of Rubisco and CCMs in seaweeds

It has previously been demonstrated that Rubisco kinetics of organisms from other phylogenetic groups have a strong correlation with CCMs, showing higher values of *k*_cat_^c^ and *K*_c_ for Rubiscos adapted to higher [CO_2_]:[O_2_] ratios (Tcherkez *et al.*, 2006; Savir *et al.*, 2010; Whitney *et al.*, 2011). The positive correlation observed in the present study between Rubisco kinetic parameters and the CCM proxies, δ^13^C_alga_ and pH compensation point, for all species analyzed together (Table 5) is in agreement with these previous reports. By contrast, when only form ID Rubiscos were analyzed, *K*_c_ was correlated with neither δ^13^C_alga_ nor the pH compensation point, although *k*_cat_^c^ was positively correlated with the pH compensation point at both assay temperatures studied. These results suggest a positive selection in form ID Rubisco *k*_cat_^c^ from macroalgae possessing CCMs without a significant concomitant increase in *K*_c_, diverging from the canonical trade-off between *K*_c_ and *k*_cat_^c^.

It is important to consider that, unlike in C_4_ plants, the expression of CCMs in algae is facultative, and is regulated by a large number of environmental factors (Giordano *et al.*, 2005). Thus, pH drift experiments must indicate the presence of a potential CCM capacity, since long-term exposure under constant and saturating irradiance to decreasing CO_2_ concentrations as the pH increases may lead to the expression of CCM components (Raven *et al.*, 2005). In contrast, δ^13^C_alga_ values might reflect CCM operation during the growth of the thalli, which could be down-regulated due to energetic constraints (Hepburn *et al.*, 2011). Despite the different timescales of both measurements, a positive correlation between δ^13^C_alga_ and pH compensation point was found, along with a striking separation between intertidal and subtidal species for these measurements (see Fig. 1); similar observations were previously reported in a global meta-analysis including 141 marine macrophyte species (Stepien, 2015).

Since δ^13^C_alga_ values could be influenced not only by the isotopic composition of the C_i_ source but also by CO_2_ leakage (Sharkey and Berry, 1985), these data should be treated with caution when used as a proxy for CCM operation. High CO_2_ leakage prevents the accumulation of δ^13^C_alga_ within the intracellular carbon pool, thereby decreasing δ^13^C_alga_, which must approach the Rubisco isotope fractionation. Moreover, Rubisco isotope fractionation of the analyzed red and brown seaweeds, as previously shown for other ID Rubiscos (Boller *et al.*, 2015), might be different from that of spinach Rubisco (–30‰; Roeske and O’Leary, 1984), even though this value has been widely used as a cutoff for excluding HCO_3_^–^ use in marine seaweeds (Maberly *et al.*, 1992; Raven *et al.*, 2002*a*). pH drift experiments could also be affected by CO_2_ leakage and proton extrusion, leading to a lower pH compensation point than the one corresponding to the species’ capacity for HCO_3_^−^ use. However, these interferences might be negligible in our study because total alkalinity was not significantly altered after pH drift experiments (data not shown). Despite the possible effect of CO_2_ leakage, most of the analyzed species showed pH compensation points significantly higher than 9, indicating that these species must possess the ability to use HCO_3_^−^ for photosynthesis.

Only the lower sublittoral rhodophytes *Phycodrys rubens* and *Ptilota gunneri* showed a pH compensation point ≤9; these species were also the only ones with a δ^13^C_alga_ value more negative than –30‰. Therefore, assuming the limitations explained above, these findings might suggest that photosynthesis in these species relies only on diffusive CO_2_ entry. Our results are in agreement with previous studies suggesting the presence or absence of CCMs in the same species as were analyzed in the present study (Surif and Raven, 1989; Johnston *et al.*, 1991; Maberly *et al.*, 1992; Beardall and Roberts, 1999; Sherlock and Raven, 2001; Raven *et al.*, 2002*a*, 2005; Klenell *et al.*, 2004; Gordillo *et al.*, 2006; Iñiguez *et al.*, 2016*b*; Olischläger *et al.*, 2017).

All polar populations had higher or similar pH compensation point and δ^13^C_alga_ values than their cold-temperate counterparts (Table 4), even though the dissolved CO_2_ concentration of cold air-equilibrated seawater is significantly higher than at warmer temperatures (Skirrow, 1975). This fact, together with the observed strong decrease in *K*_c_ and *k*_cat_^c^ at 4 °C, would lead to closer CO_2_ saturation conditions of the analyzed form ID Rubiscos in polar air-equilibrated seawater, although Rubisco oxygenation kinetics must be analyzed in these species to further corroborate this assumption. Therefore, the maintenance or even increase of active HCO_3_^−^ use at low temperatures might be related to the fact that equilibrium conditions are not frequently met in cold oceans owing to the thermohaline circulation, biological activity, and slow equilibration of CO_2_ between the surface of the oceans and the atmosphere relative to that between CO_2_ and the other DIC species (Raven and Falkowski, 1999). The solubility of O_2_ also increases at low temperatures (Skirrow, 1975), and there is a considerable reduction in the uncatalyzed rate of CO_2_ supply from bicarbonate (Egleston *et al.*, 2010) and the diffusion rate of CO_2_ (Boudreau, 1997) in cold waters. Furthermore, the exposure to continuous light during the summer months at polar latitudes in combination with low temperatures results in the activation of photoprotection mechanisms for dissipation of excess energy; one example is the relevant level of cyclic electron flow reported for Antarctic diatoms (Goldman *et al.*, 2015), so the energetically costly CCMs might be part of these photoprotection mechanisms at low temperatures (Gordillo *et al.*, 2016). It should be taken into account that the ability to use HCO_3_^−^ for photosynthesis does not necessarily mean that C_c_ in steady-state photosynthesis is higher than [CO_2_] in the external medium. C_i_ uptake can operate at a lower rate than that of Rubisco carboxylation, yet still improve CO_2_ ﬁxation by lessening the degree to which the [CO_2_] limits Rubisco carboxylation (Koch *et al.*, 2013).

The negative correlation between [Rubisco]/[TSP] and *k*_cat_^c^ that was obtained for all the species analyzed together (Table 5) agrees well with previous studies indicating that faster carboxylation rates of Rubisco enable these seaweeds to invest less in Rubisco relative to the total soluble protein fraction (Seemann *et al.*, 1984; Ghannoum *et al.*, 2005; Galmés *et al.*, 2014). The same trend was observed when only form ID Rubiscos were considered, although it was not statistically significant (*P*=0.054 for 25 °C, *P*=0.059 for 4 °C). Nevertheless, [Rubisco]/[TSP] must be higher in actively growing thalli than in old specimens, whereas Rubisco kinetics are constant for a particular organism, which might alter the previous correlation. In the present study, the growth rate of field samples was unknown, although young thalli were selected for the analyses when possible.

The absence of correlation between the two CCM proxies, δ^13^C_alga_ and pH compensation point, and [Rubisco]/[TSP] (*R*=–0.037, *P*=0.893 and *R*=–0.444, *P*=0.128, respectively) contrasts with previous results found in comparisons of C_3_ and C_4_ plants that indicated a lower [Rubisco]/[TSP] in C_4_ plants owing to the contribution of proteins involved in CCMs to total soluble protein and less investment in Rubisco (Ghannoum *et al.*, 2005). However, in algae, many CCM proteins are insoluble membrane transporters, and soluble carbonic anhydrases might be also present in organisms without CCMs (Beer *et al.*, 2014). Remarkably, brown seaweeds were found to have 3-fold to 4-fold higher [Rubisco]/[TSP] than red and green macroalgae (Tables 2 and 3), which could be related to the high productivity of Laminariales and Desmarestiales underwater forests in cold-temperate to polar waters (Wiencke *et al.*, 2007). Alternatively, differences in [Rubisco]/[TSP] between groups could be partly related to a lower efficiency in the extraction of proteins other than Rubisco in the studied brown seaweeds, due to the presence of high contents of secondary metabolites and polysaccharides in these species that might interfere with protein solubilization. Very efficient total protein extraction protocols have been developed for Laminariales (Olischläger *et al.*, 2014), but these protocols unavoidably involve protein denaturation.

### Intraspecific adaptation of seaweed Rubiscos to low temperatures

The Arctic populations of *Palmaria palmata* and *S. latissima* presented significantly higher *k*_cat_^c^/*K*_c_ at 4 °C than their temperate counterparts, driven by an increase in *k*_cat_^c^ (Table 3). This would lead to higher CO_2_-saturated photosynthetic rates at low temperatures in the polar populations compared with the cold-temperate populations. Similarly, when comparing populations belonging to the genus *Palmaria*, it was observed that the endemic Antarctic species *Palmaria decipiens* showed significantly higher *k*_cat_^c^ and *k*_cat_^c^/*K*_c_ at 4 °C than the Arctic *Palmaria palmata* (Table 2), which might be related to the much longer cold-water history of Antarctica relative to the Arctic Ocean (Zacher *et al.*, 2011).

Despite these differences in Rubisco kinetics at 4 °C between polar and temperate populations, and considering their similar [Rubisco]/[TSP] values (Table 3), we suspect the existence of a higher Rubisco activation state in polar red and brown seaweeds in order to achieve the photosynthetic rates that have been measured in previous studies of the same species and locations (Thomas and Wiencke, 1991; Iñiguez *et al.*, 2016*a*, *b*; Olischläger *et al.*, 2017). Young *et al.* (2015) also suggested that Rubisco must be almost fully active in Antarctic diatoms, after comparison of its photosynthetic carbon fixation rates with Rubisco *k*_cat_^c^ and quantity at low temperatures.

In contrast, the polar populations of the chlorophyte *A. arcta* showed no significant differences in *k*_cat_^c^/*K*_c_ at 4 °C compared with the temperate population, although a more than 2-fold increase in the [Rubisco]/[TSP] of both polar populations was observed (Table 3). These results are in agreement with those reported by Devos *et al.* (1998), who observed a similar 2-fold increase in the relative Rubisco content of two psychrophilic strains of the chlorophyte genus *Chloromonas* compared with their mesophilic counterparts, which might suggest different photosynthetic cold-adaptation responses between form IB and form ID Rubiscos.

The Arctic population of *Phycodrys rubens* did not show cold-adaptive traits compared with the cold-temperate population of this species, in terms of either Rubisco kinetics or [Rubisco]/[TSP]. As this species grows in the lower sublittoral, its photosynthetic rates should be constrained by the low irradiance reaching these depths and not by the maximum Rubisco carboxylation rate, probably leading to a lack of energetically expensive CCMs (Raven *et al.*, 2002*a*, *b*, 2014), as suggested by the CCM proxies (Table 4).

It is unsurprising that the (*K*_c_)^25 °C^/(*K*_c_)^4 °C^ ratio of polar Rubiscos was not lower (i.e. *K*_c_ less temperature dependent) than that of their cold-temperate counterparts (Table 3), as all Rubiscos, from either polar or temperate populations, showed increasing affinity for CO_2_ at decreasing temperatures at the expense of a reduced catalytic activity, which facilitates the CO_2_ saturation of the enzyme. Thus, assuming that carbon fixation might constrain photosynthesis in cold waters, polar environments should lead to a selection for higher *k*_cat_^c^ Rubiscos instead of lower *K*_c_. However, this assumption does not take into account biochemical processes other than Rubisco activity that can be affected by temperature, such as RuBP regeneration (Bernacchi *et al.*, 2003). Our results showed that the ratios (*k*_cat_^c^)^25 °C^/(*k*_cat_^c^)^4 °C^ and (*k*_cat_^c^/K_c_)^25 °C^/(*k*_cat_^c^/K_c_)^4 °C^ were significantly lower in the Arctic *S. latissima* compared with its cold-temperate counterpart, but not in the other polar versus temperate population comparisons, while the lowest values of (*k*_cat_^c^)^25 °C^/(*k*_cat_^c^)^4 °C^ within the Rhodophyta and Ochrophyta were obtained for the polar endemic species analyzed from each phylum (Table 2). This might reflect adaptation of the enzyme’s temperature sensitivity according to its environment, as previously described (Sage, 2002; Galmés *et al.*, 2005, 2015, 2016; Young *et al.*, 2015).

### Conclusions

Our results provide novel data on Rubisco kinetics from ecologically relevant polar and cold-temperate seaweeds belonging to different taxonomic groups. In contrast to previous findings in other photosynthetic groups, there was no correlation between either *K*_c_ or [Rubisco]/[TSP] and the CCM proxies for the red and brown seaweeds analyzed in the present study. Moreover, most of the analyzed polar populations showed signs of CCMs despite the lower *K*_c_ in form ID seaweed Rubiscos and the higher dissolved [CO_2_] in air-equilibrated seawater at 4 °C. We also report evidence of cold adaptation in Rubisco carboxylation kinetics or [Rubisco]/[TSP] of polar macroalgae likely possessing CCMs. In spite of this, photosynthesis at low temperatures and saturating irradiance conditions in red and brown seaweeds must be constrained by carbon fixation rates, and must require a high Rubisco activation state. Further studies of the regulation of form ID Rubiscos (see Mueller-Cajar *et al.*, 2011; Loganathan *et al.*, 2016) in seaweeds, about which data are currently scarce, are needed in order to make accurate predictions of productivity and ecosystem functioning in near-future scenarios.

## Supplementary data

Supplementary data are available at *JXB* online.

Table S1. Rubisco carboxylation kinetics at 25ºC taken from other datasets and used in Fig. 3.

## Supplementary Material

Supplementary_Table_S1
