# Supplementary material for: Rubisco carboxylation kinetics and inorganic carbon utilization in polar versus cold-temperate seaweeds
Source: J Exp Bot. 2018 Dec 21;70(4):1283–97. doi: 10.1093/jxb/ery443 (PMC6382342; doi:10.1093/jxb/ery443)
Supplement: Supplementary_Table_S1 [file ery443_suppl_supplementary_table_s1.pdf]

Supplementary data

Table S1. Form I Rubisco carboxylation kinetics at 25ºC taken from other datasets and used in Figure 3; n.d., not determined.

	K_c (μM)	k_{cat}^c (s^{-1})	k_{cat}^c/K_c (s^{-1} mM^{-1})	References
**Rhodophyta**				
**Cyanidiophyceae**				
Cyanidium caldarium	7	1.3	186	Badger *et al.*, 1998
Cyanidium partita	7	1.6	229	Badger *et al.*, 1998
Galdieria sulfuraria	3	1.2	364	Whitney *et al.*, 2001
**Florideophyceae**				
Griffithsia monilis	9	2.6	280	Whitney *et al.*, 2001
**Ochrophyta**				
**Phaeophyceae**				
Laminaria hyperborea	12	n.d.	n.d.	Kuppers and Weidner, 1980
**Bacillariophyceae (diatoms)**				
Bellerochea cf. horologicalis	50	2.1	41	Young *et al.*, 2016
Chaetoceros calcitrans	25	2.6	103	Young *et al.*, 2016
Chaetoceros muelleri	23	2.4	104	Young *et al.*, 2016
Cylindrotheca fusiformis	n.d.	3.7	n.d.	Young *et al.*, 2016
Fragilariopsis cylindrus	64	3.5	55	Young *et al.*, 2016
Phaeodactylum tricornutum	41	3.3	81	Young *et al.*, 2016
Skeletonema marinoi	68	3.2	47	Young *et al.*, 2016
Thalassiosira oceanica	65	2.4	36	Young *et al.*, 2016
Thalassiosira weissflogii	65	3.2	49	Young *et al.*, 2016
**Haptophyta**				
Pavlova lutheri	14	2.5	172	Heureux *et al.*, 2017
Pleurochrysis carterae	18	3.3	186	Heureux *et al.*, 2017
Tisochrysis lutea	24	2.2	91	Heureux *et al.*, 2017
**Chlorophyta**				
Chlamydomonas reinhardtii	31	2.1	68	Genkov *et al.*, 2010
Codium fragile	43	n.d.	n.d.	Yeoh *et al.*, 1981
Scenedesmus obliquus	38	n.d.	n.d.	Jordan and Ogren, 1981
Ulva sp.	70	n.d.	n.d.	Yeoh *et al.*, 1981

**C_3 higher plants**				
Arabidopsis thaliana	14	3.2	222	Whitney *et al*., 2011
Avena sativa	11	2.3	213	Hermida-Carrera *et al*., 2016
Beta vulgaris	11	2.0	185	Hermida-Carrera *et al*., 2016
Brassica oleracea	12	2.1	178	Hermida-Carrera *et al*., 2016
Capsicum annuum	10	1.9	198	Hermida-Carrera *et al*., 2016
Chenopodium alba	11	2.9	259	Kubien *et al*., 2008
Coffea Arabica	11	2.1	191	Hermida-Carrera *et al*., 2016
Crithmum maritimum	9	3.4	378	Galmés *et al*., 2014
Cucurbita maxima	9	2.2	244	Hermida-Carrera *et al*., 2016
Eucalyptus neglecta	8	2.5	312	Galmés *et al*., 2014
Flaveria cronquistii	11	3.1	287	Kubien *et al*., 2008
Glycine max	9	1.5	174	Hermida-Carrera *et al*., 2016
Hordeum vulgare	9	2.4	267	Hermida-Carrera *et al*., 2016
Ipomoea batatas	12	2.5	208	Hermida-Carrera *et al*., 2016
Lactuca sativa	11	2.2	198	Hermida-Carrera *et al*., 2016
Limonium gibertii	8	4.6	561	Parry *et al*., 2007
Manihot esculenta	6	1.4	229	Hermida-Carrera *et al*., 2016
Medicago sativa	10	1.7	175	Hermida-Carrera *et al*., 2016
Nicotiana tabacum	11	3.4	309	Whitney *et al*., 1999
Oryza sativa	8	2.1	262	Hermida-Carrera *et al*., 2016
Phaseolus vulgaris	8	1.7	218	Hermida-Carrera *et al*., 2016
Solanum lycopersicum	10	2.3	237	Hermida-Carrera *et al*., 2016
Spinacia oleracea	12	3.2	265	Kubien *et al*., 2008
Triticum aestivum	11	2.7	248	Carmo-Silva *et al*., 2010
Teucrium heterophyllum	7	2.7	386	Galmés *et al*., 2014
**C_4 higher plants**				
Amaranthus edulis	18	4.1	225	Kubien *et al*., 2008
Amaranthus hybridus	16	3.8	237	Jordan and Ogren, 1981
Cynodon dactylon	21	3.7	176	Carmo-Silva *et al*., 2010
Flaveria australasica	22	3.8	173	Kubien *et al*., 2008
Flaveria bidentis	20	4.2	208	Kubien *et al*., 2008
Flaveria kochiana	23	3.7	163	Kubien *et al*., 2008
Flaveria trinervia	18	4.4	246	Kubien *et al*., 2008
Paspalum dilatatum	20	3.4	171	Carmo-Silva *et al*., 2010
Saccharum officinarum	26	3.9	148	Hermida-Carrera *et al*., 2016
Sorghum bicolor	30	5.4	180	Sage and Seemann, 1993
Zea mays	32	4.1	130	Hermida-Carrera *et al*., 2016
Zoysia japonica	18	4.1	222	Carmo-Silva *et al*., 2010

References

Badger MRT, Andrews J, Whitney SM, Ludwig M, Yellowlees DC, Leggat W, Price GD. 1998. The diversity and coevolution of Rubisco, plastids, pyrenoids, and chloroplast-based CO_2-concentrating mechanisms in algae. Canadian Journal of Botany **76,** 1052–1071.

Carmo-Silva AE, Keys AJ, Andralojc PJ, Powers SJ, Arrabaca MC, Parry MAJ. 2010. Rubisco activities, properties, and regulation in three different C_4 grasses under drought. Journal of Experimental Botany **61,** 2355–2366.

Galmés J, Kapralov MV, Andralojc PJ, Conesa MÀ, Keys AJ, Parry MAJ, Flexas J. 2014. Expanding knowledge of the Rubisco kinetics variability in plant species: environmental and evolutionary trends. Plant, Cell & Environment **37,** 1989–2001.

Genkov T, Meyer M, Griffiths H, Spreitzer RJ. 2010. Functional Hybrid Rubisco Enzymes with Plant Small Subunits and Algal Large Subunits engineered rbcS cDNA for expression in *Chlamydomonas*. Journal of Biological Chemistry **285,** 19833–19841.

Hermida-Carrera C, Kapralov MV, Galmés J. 2016. Rubisco catalytic properties and temperature response in crops. Plant Physiology **171,** 2549–2561.

Heureux AM, Young JN, Whitney SM, Eason-Hubbard MR, Lee RBY, Sharwood RE, Rickaby REM. 2017. The role of Rubisco kinetics and pyrenoid morphology in shaping the CO_2-concentrating mechanisms of Haptophyte microalgae. Journal of Experimental Botany **68,** 3959–3969.

Jordan DB, Ogren WL. 1981. Species variation in the specificity of ribulose bisphosphate carboxylase/oxygenase. Nature **291,** 513–515.

Kubien DS, Whitney SM, Moore PV, Jesson LK. 2008. The biochemistry of Rubisco in *Flaveria*. Journal of Experimental Botany **59,** 1767–1777.

Kuppers U, Weidner M. 1980. Seasonal-variation of enzyme activities in *Laminaria hyperborea*. Planta **148,** 222–230.

Parry MAJ, Madgwick PJ, Carvalho JFC, Andralojc PJ. 2007. Prospects for increasing photosynthesis by overcoming the limitations of Rubisco. Journal of Agricultural Science **145,** 31–43.

Sage RF, Seemann JR. 1993. Regulation of ribulose-1,5-bisphosphate carboxylase/oxygenase activity in response to reduced light intensity in C_4 plants. Plant Physiology **102,** 21–28.

Whitney SM, Baldet P, Hudson GS, Andrews TJ. 2001. Form I Rubiscos from non-green algae are expressed abundantly but not assembled in tobacco chloroplasts. The Plant Journal **26,** 535–547.

Whitney SM, Houtz RL, Alonso H. 2011. Advancing our understanding and capacity to engineer nature's CO_2-sequestering enzyme, Rubisco. Plant Physiology **155,** 27–35.

Whitney SM, von Caemmerer S, Hudson GS, Andrews TJ. 1999. Directed mutation of the Rubisco large subunit of tobacco influences photorespiration and growth. Plant Physiology **121,** 579–588.

Yeoh HH, Badger MR, Watson L. 1981. Variations in kinetic-properties of ribulose-1,5-bisphosphate carboxylases among plants. Plant Physiology **67,** 1151–1155.

Young JN, Heureux AMC, Sharwood RE, Rickaby REM, Morel FMM, Whitney SM. 2016. Large variation in the Rubisco kinetics of diatoms reveals diversity among their carbon-concentrating mechanisms. Journal of Experimental Botany **67,** 3445–3456.
